# Hemodiafiltration Attenuates NETosis Compared With High-Flux Hemodialysis in End-Stage Kidney Disease Patients

**DOI:** 10.1016/j.ekir.2025.06.002

**Published:** 2025-06-10

**Authors:** Lital Remez-Gabay, Olga Vdovich, Faten Y. Andrawes Barbara, George Jiries, Etty Kruzel-Davila

**Affiliations:** 1 Department of Nephrology, Galilee Medical Center, Nahariya, Isreal; 2 Azrieli Faculty of Medicine, Bar-Ilan University, Zefat, Isreal; 3 Nephrology Laboratory, Research Institute of Galilee Medical Center, Nahariya, Isreal

**Keywords:** chronic kidney disease (CKD), diabetes mellitus (DM), hemodiafiltration (HDF), high- flux hemodialysis (HFHD), neutrophils extracellular tramp (NETosis)

## Abstract

**Introduction:**

Patients with chronic kidney disease (CKD) and diabetes mellitus face a heightened risk of cardiovascular complications and infections, potentially exacerbated by dysregulated NETosis. Given the superior survival rates observed with hemodiafiltration (HDF) over high-flux hemodialysis (HD; HFHD) and the documented NETosis dysregulation in HD and in patients with diabetes mellitus, this study aimed to investigate the impact of dialysis modality on NETosis activity in patients on HD, stratified by diabetes status.

**Methods:**

A total of 20 patients on HD (10 with diabetes, 10 without diabetes) undergoing HDF treatment were recruited. Blood samples were collected before and after HDF. After transition to HFHD treatment, blood samples were taken again after 1 and 3 weeks of HDFD treatment. Neutrophils were isolated, stimulated with phorbol-12-myristate-13-acetate, and stained for the following NETosis markers: peptidyl arginine deiminase 4 (PAD4), neutrophil elastase (NE), myeloperoxidase (MPO), histone H3, and double-stranded DNA (dsDNA). Data were acquired using a flow cytometer. In addition, serum levels of citrullinated histone H3 (citH3), MPO, and NE were measured using enzyme-linked immunosorbent assay.

**Results:**

Our results demonstrate a significant increase in NETosis activation and markers after HFHD treatment compared with HDF treatment. NETosis markers significantly increased in serum after 3 weeks of HFHD treatment. In addition, significantly lower NETosis markers were observed in patients with diabetes than in patients without diabetes.

**Conclusion:**

The increase in NETosis markers after 3 weeks of HFHD compared with HDF highlights the role of HDF in mitigating dysregulated NETosis. Further research is needed to explore differences in NETosis profiles across patient populations and assess their clinical implications based on dialysis modality.

## Graphical abstract

**Figure:**
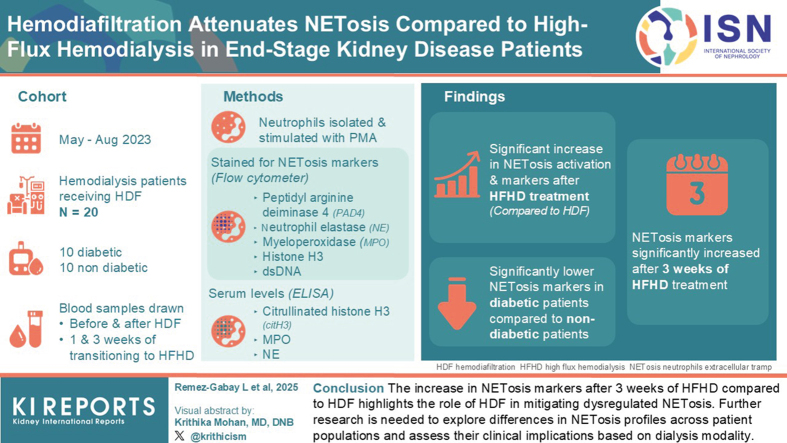

CKD poses a major global public health challenge, impacting approximately 850 million individuals worldwide. Many of these patients progress to end-stage kidney disease, requiring renal replacement therapies such as HD, where such treatments are accessible. Patients with CKD face not only the burden of declining renal function but also a high prevalence of comorbidities and complications, particularly cardiovascular diseases and infections.^1^, ^2^, ^3^ Cardiovascular disease remains the leading cause of morbidity and mortality in this population, accounting for > 40% of deaths among patients on dialysis. This elevated risk is driven by both traditional cardiovascular risk factors, such as hypertension and diabetes and CKD-specific factors, including accumulation of uremic toxins, fluid overload, and systemic inflammation.^1^, ^2^, ^3^ In addition to cardiovascular disease, patients with CKD undergoing HD are highly susceptible to infections, which contribute significantly to morbidity and mortality. This vulnerability is partly due to secondary immunodeficiency related to kidney disease, frequent vascular access, and associated comorbidities.^1^^,^^4^ Uremic toxins impair multiple immune functions, leading to reduced neutrophil activity; impaired cytokine release; and dysfunction of natural killer, T, and B cells. Chronic low-grade inflammation in uremic patients suppresses the immune response during infections, contributing to poor outcomes.^4^ A key process in CKD-related inflammation is dysregulated NETosis, a form of neutrophil extracellular trap (NET) formation. NETosis is an evolutionarily conserved process aimed at entrapping microorganisms. Neutrophils form these NETs by releasing decondensed chromatin (DNA coiled around histones) lined with the content of neutrophil intracytoplasmic granules.^5^ *In vitro*, NETosis can be induced by phorbol-12-myristate-13-acetate and requires activation of the Raf-MEK-ERK pathway along with reduced NAD phosphate oxidase-dependent production of reactive oxygen species. An increase in cytosolic calcium ions activates reduced NAD phosphate oxidase and functions as a cofactor for PAD4, which catalyzes the citrullination of histone H3, leading to chromatin decondensation.^6^ This process results in the extrusion of a mixture of DNA and bactericidal proteins, including MPO and NE and citH3, all of which serve as markers of NETosis.^6^^,^^7^ In addition to phorbol-12-myristate-13-acetate, NETosis can also be triggered by antibodies, proinflammatory cytokines, chemokines, and sterile inflammatory stimuli such as high glucose levels, cholesterol, complement component C5a, and hypoxia.^6^^,^^7^ Therefore, NETosis exhibits a dual-edged nature. Although it was initially considered a protective host defense mechanism against pathogens, uncontrolled NET formation can lead to significant tissue damage; thus, promoting necroinflammation. This switch from a beneficial response to a harmful one contributes to cardiovascular complications and a hypercoagulable state, particularly in patients undergoing HD. Dysregulated NETosis, potentially driven by bioincompatibility during HD, is thought to contribute to the significant comorbid burden observed in this patient population.^7^, ^8^, ^9^, ^10^, ^11^, ^12^ Given the high cardiovascular and infection-related morbidity and mortality in patients with end-stage kidney disease undergoing HD, there is an urgent need to explore more biocompatible dialysis modalities. HDF has emerged as a superior alternative to conventional HFHD. By combining diffusion and convection, HDF provides enhanced removal of larger uremic toxins implicated in inflammation and cardiovascular events.^13^^,^^14^ Recent studies suggest that HDF may reduce cardiovascular morbidity, infection rates, and improved survival, offering potential improvements in survival and quality of life for patients with CKD.^15^, ^16^, ^17^

In light of the deleterious effects of enhanced NETosis during HD, we hypothesize that attenuated NETosis may contribute to the protective effects of HDF compared with HFHD. The combination of diffusion and convection in HDF is thought to mitigate oxidative stress and improve hemodynamic stability, leading to decreased NET formation during dialysis. This reduction in NETosis may improve immune function and reduce the risk of complications, ultimately enhancing survival outcomes in patients receiving HDF.

## Methods

### Study Design and Participants

Twenty patients on HD who had been receiving HDF with high-flux dialyzers (FX 100, Fresenius or 19H, Elisio) for ≥ 3 months were enrolled in the study between May and August 2023. Among them, 10 patients were diagnosed with type 2 diabetes mellitus and 10 were without diabetes. To ensure that baseline measurements of NETosis markers reflected a stable and physiologically uniform state, all participants began the study under their ongoing HDF regimen. This approach minimized interindividual variability and mitigated potential confounding effects associated with previous exposure to different dialysis modalities. Subsequently, all patients were transitioned to HFHD, enabling a within-subject comparison to assess the impact of dialysis modality on NETosis activity over time.

In addition, 10 healthy individuals were recruited to serve as a control group. However, the primary objective of this study was to characterize NETosis activation in response to dialysis treatment; specifically, the changes in NETosis markers before and after dialysis sessions. Therefore, the healthy individuals were used as a qualitative reference for NETosis marker levels in nondialysis individuals.

Participants were excluded if they had a history of autoimmune diseases, malignancies, chronic hepatitis B, hepatitis C, or HIV infection, or were taking medications known to directly affect the immune system. All participants provided written informed consent. The study was approved by the Helsinki Committee at the Galilee Medical Center (Approval Number: 108-22-NHR).

### Blood Samples and Neutrophil Isolation

Blood samples (ethylenediamine tetraacetic acid tubes) and serum (clot activator tubes) were collected at 2 defined time points during both the HDF and HFHD phases as follows:

(i) before dialysis, immediately before the start of the treatment session, to ensure that measurements reflected a stable predialysis baseline and were not influenced by dialysis-related changes; and (ii) after dialysis, at the end of treatment session, to assess the acute effect of the dialysis procedure on NETosis markers. This approach allowed us to directly compare within-session changes in NETosis for each dialysis modality.

After transitioning to HFHD, blood samples were collected in the same manner 1 week after HFHD treatment, with additional serum samples collected 3 weeks after HFHD treatment.

Neutrophils were purified from whole blood samples using the EasySep Direct Human Neutrophils Isolation Kit (STEMCELL Technologies), according to the manufacturer’s instruction.

### NETosis Assay

Purified neutrophils (1 × 10^6^/ml) were seeded in a 24-well plate and incubated with RPMI 1640 medium (Sartorius) containing 5% FBS HI (Gibco Fisher Scientific) at 37 °C and 5% CO_2_. To assess NETosis, neutrophils were stimulated with 100 nM phorbol-12-myristate-13-acetate (P8139; Sigma-Aldrich) for 1 hour or left unstimulated.

Following stimulation, neutrophils were collected and washed 3 times with staining buffer (phosphate-buffered saline containing 1% fetal bovine serum HI). Neutrophils underwent a standard cell staining protocol and incubated with the following 2 separated mixes: (i) AF647 anti-human PAD4 (sc-365369; Santa Cruz Biotechnology) and FITC anti-human MPO (ab11729, Abcam) and (ii) PE anti-human NE (BD568908; BD Biosciences) and AF647 anti-human-Histone H3 (ab207543; Abcam). After 3 washes, 7-amino-actinomycin D (7AAD) viability dye for dsDNA staining (A07704; Beckman Coulter International) was added to each mix. Data acquisition was performed using the Navios Flow Cytometer (Beckman Coulter) and analyzed with Kaluza software version 2.1 (Beckman Coulter) (Supplementary Figure S1).

### NETosis Markers in Serum

All serum samples were diluted 1:2 and citH3 was quantified using the citH3 enzyme-linked immunosorbent assay kit (501620; Cayman Chemical). Serum was diluted 1:1000 for MPO measurement using MPO enzyme-linked immunosorbent assay kit (501410; Cayman Chemical). NE was detected in serum (1:500 dilution) using Human NE enzyme-linked immunosorbent assay kit (ab204730; Abcam) according to the manufacturer’s instructions.

### Statistical Analysis

The clinical and demographic data of patients with diabetes and those without diabetes were analyzed statistically. Quantitative variables were compared between the groups using the Mann-Whitney U test, whereas categorical variables were analyzed using Fisher exact test. Paired comparisons between HDF and HFHD parameters, stratified by diabetes status, were conducted using the Wilcoxon signed ranks test.

For NETosis assays, statistical analyses and graph generation were performed using Prism software version 2.1. All conditions were compared using paired *t* tests, or Wilcoxon tests for nonparametric data. Differences between groups in the patients with diabetes and those without diabetes were assessed using unpaired *t* tests. Statistical significance was defined as *P* < 0.05. Data are presented as mean ± SEM.

## Results

### Patients Demographics and Baseline Characteristics

Baseline clinical and laboratory characteristics of patients with diabetes and those without diabetes are summarized in Table 1. The average age of patients with diabetes was 60.7 years (± 5.12), whereas patients without diabetes had a slightly younger average age of 58.5 years (± 12) (*P* = 0.9). Patients with diabetes exhibited a trend toward a shorter HD vintage, with a median of 4.27 years (interquartile range: 2.72–6.75) compared with 5.2 years (interquartile range: 4.37–10) in patients without diabetes (*P* = 0.06). Both groups had similar median body weights, with patients with diabetes weighing 89.75 kg and those without diabetes weighing 85.5 kg (*P* = 0.8). The majority of patients in both groups were treated via tunneled catheter (60% of those with diabetes and 50% of those without diabetes), with no significant difference in vascular access types (tunneled catheter, arteriovenous fistula, arteriovenous graft). Patients with diabetes had a lower median convection volume per session (18 L, interquartile range: 18–21) than those without diabetes (20.5 L, interquartile range: 19.75–21.25) (*P* = 0.06).

**Table 1:** Baseline clinical and laboratory characteristics of patients with diabetes and those without diabetes

Characteristic	Patients without diabetes *n* = 10	Patients with diabetes *n* = 10	*P*-value
Age, mean (SD)	58 (12)	60 (5.12)	0.9^a^
Female, *n* (%)	5 (50)	6 (60)	1^b^
HD vintage (yrs), median (IQR)	5.2 (4.37–10)	4.27 (2.72–6.75)	0.06^a^
Weight (Kg.), Median (IQR)	85.5 (74.87–92.62)	89.75 (73.75–94.12)	.5^a^
BMI > 30 kg/m^2^, *n* (%)	5 (50)	8 (80)	0.4^b^
Tunneled catheter, *n* (%)	5 (50)	6 (60)	1^b^
AVF, *n* (%)	5 (50)	3 (30)	
AVG, *n* (%)	0 (0)	1 (10)	
Convection volume (l/session), median (IQR)	20.5 (19.75–21.25)	18 (18–21)	0.06^a^
Blood pressure, *n* (%)	8 (80)	9 (90)	1^b^
Congestive heart failure, *n* (%)	4 (40)	10 (100)	0.01^b^
Ischemic heart disease, *n* (%)	1 (10)	9 (90)	0.001^b^
Atrial fibrillation, *n* (%)	1 (10)	3 (30)	0.6^b^
Hyperlipidemia, *n* (%)	8 (80)	10 (100)	0.5^b^
S/P cerebrovascular accident, *n* (%)	1 (10)	1 (10)	1^b^
ADPKD, *n* (%)	4 (40)	1 (10)	0.3^b^
Cigarette smoking, *n* (%)	0 (0)	3 (30)	0.2^b^
KT/V SP, median (IQR)	1.49 (1.29–1.57)	1.51 (1.36–1.61)	0.6^a^
HbA1c (%), mean (SD)	4 (0.36)	7 (1.67)	< 0.001^a^
DM duration (yr), median (IQR)	---	15.5 (11–23)	
ACEI, *n* (%)	1 (10)	1 (10)	1^b^
ARB, *n* (%)	2 (20)	4 (40)	0.6^b^
CCB, *n* (%)	6 (60)	6 (60)	1^b^
BB, *n* (%)	6 (60)	9 (90)	0.3^b^
Aspirin, *n* (%)	6 (60)	7 (70)	1^b^
Plavix, *n* (%)	0 (0)	2 (20)	0.5^b^
Insulin, *n* (%)	--	5 (50)	
Sitagliptin, *n* (%)	--	1 (10)	
Hb (g/dl), median (IQR)	10.6 (9.95–11.3)	11.7 (10.77–12.45)	0.06^a^
WBC (10^3^/μl), median (IQR)	5.22 (4.76–6.38)	7.3 (6.7–8.49)	0.005^a^
Neutrophils %, median (IQR)	64 (49.17–67.92)	71 (68.6–72.92)	0.01^a^
Monocytes %, median (IQR)	8 (6.95–11.02)	7 (5.9–9.4)	0.5^a^
Eosinophils %, median (IQR)	2 (1.65–6.92)	2 (1.70–4.05)	0.5^a^
Lymphocytes %, median (IQR)	19 (16.87–26.8)	18 (15.62–23.15)	0.5^a^
Platelets (10^3^/μl), median (IQR)	178.5 (148–239.25)	205.5 (157.25–255)	0.4^a^
CRP (mg/dl), median (IQR)	6.15 (3.52–27.27)	8.5 (3.35–22.77)	0.8^a^
Albumin (g/dl), median (IQR)	4 (3.67–4.11)	3.87 (3.62–4.2)	0.8^a^
Ferritin (μg/l), median (IQR)	364 (259.94–473.9)	443.08 (253.72–590.5)	0.6^a^
PTH (pg/ml), median (IQR)	507.35 (318–824.03)	582.56 (355.06–877.5)	0.7^a^

ACEI, angiotensin-converting enzyme inhibitor; ADPKD, autosomal polycystic kidney disease; ARB, angiotensin receptor blocker; AVF, arteriovenous fistula; AVG, arteriovenous graft; BB, beta blockers; BMI, body mass index; CCB, calcium channel blocker; CRP, C-reactive protein; DM, diabetes mellitus; Hb, hemoglobin; HD, hemodialysis; IQR, interquartile range; KT/V SP, dialysis dose (K – Dialyzer clearance of urea (ml/min) t – Dialysis time (minutes) V – Volume of distribution of urea); PTH, parathyroid hormone; S/P, status post; WBC, white blood cells.

a Mann-Whitney test.

b Fisher exact test.

A notable disparity was observed in cardiovascular comorbidities. All patients with diabetes (100%) had congestive heart failure, compared with 40% of patients without diabetes (*P* = 0.01). Similarly, ischemic heart disease was significantly more prevalent in patients with diabetes (90 %) than in patients without diabetes (10%) (*P* = 0.001). As expected, patients with diabetes had significantly higher median HbA1c levels (7.84%) than those without diabetes (4.87%) (*P* < 0.001).

Patients with diabetes showed a trend toward higher hemoglobin levels (median: 11.75 g/dl) than those without diabetes (median: 10.6 g/dl, *P* = 0.06). Furthermore, they exhibited significantly elevated white blood cell counts and higher neutrophil percentages than those without diabetes (*P* = 0.005, 0.01, respectively). No significant differences were observed between the 2 groups in terms of antihypertensive or antiaggregation medications, as well as other hematological, inflammatory, or endocrine markers, including eosinophils, monocytes, lymphocytes, platelets, C-reactive protein, albumin, ferritin, and parathyroid hormone levels (Table 1).

Overall, this comparison highlights the greater prevalence of ischemic heart disease, and congestive heart failure in patients with diabetes, who also demonstrated higher white blood cell counts. Furthermore, patients with diabetes had shorter HD vintages and lower convection volumes during HDF sessions, emphasizing potential differences in dialysis treatment characteristics that could influence NETosis.

### Significant Increase in NETosis Markers in Stimulated Neutrophils After HFHD Treatment Compared With HDF in Both Patients With Diabetes and Those Without Diabetes

To investigate the impact of HD modality on NETosis, we recruited 20 patients undergoing HDF as their prescribed treatment (10 with diabetes and 10 without diabetes). Blood samples were obtained from all participants before and after HDF treatment. Subsequently, HDF treatment was switched to HFHD, and blood samples were collected again in the same manner after 1 week of HFHD treatment. Key dialysis parameters, including treatment time, body weight, weight loss, ultrafiltration rate, blood flow, dialysate flow, and fluid or hemodynamic status were not significantly different between the HDF or HFHD groups and are presented in Supplementary Table S1.

**Figure 1:**
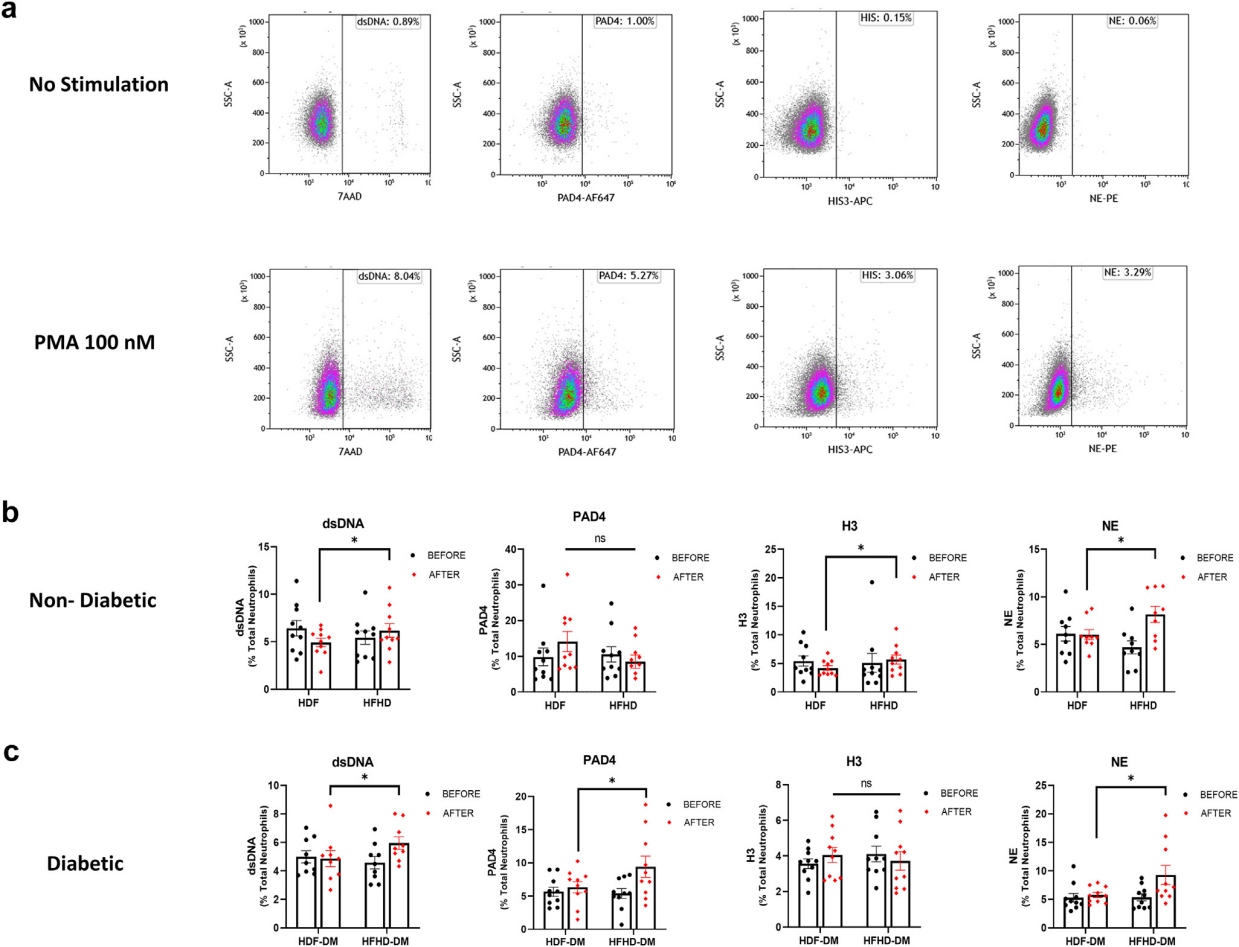
Significant increase in NETosis markers in stimulated neutrophils following HFHD treatment compared to HDF in both patients with diabetes and those without diabetes. (a) Flow cytometry quantification of NETosis markers in neutrophils, both unstimulated and stimulated with 100 nM PMA for 1 hour. Stimulation led to increased expression of NETosis markers including viability dye dsDNA-7-AAD, PAD4-AF647, histone H3-APC, and neutrophil elastase (NE-PE). (b) NETosis markers in patients without diabetes before and after HDF or HFHD treatment. Significant increases in dsDNA, H3, and NE were observed following HFHD compared with HDF. No significant change was seen in PAD4. (c) NETosis markers in patients with diabetes before and after HDF or HFHD treatment. Significant increases were noted in dsDNA, PAD4, and NE after HFHD compared with HDF. H3 levels did not differ significantly. Data are presented as mean ± SEM. All statistical analysis was performed using paired *t* test, ∗*P* < 0.05. H3, histone H3; HDF, hemodiafiltration; HFHD, high-flux hemodialysis; NE, neutrophil elastase; PAD4, peptidyl arginine deiminase 4; PMA, phorbol-12-myristate-13-acetate.

All samples were collected for neutrophil purification to undergo NETosis assays. All NETosis markers were elevated after cell stimulation (Figure 1a). Our results demonstrated a significant increase in NETosis markers after HFHD treatment compared with HDF treatment. Patients without diabetes showed a significant increase in the following markers: dsDNA (*P* = 0.04, *n* = 10), H3 (*P* = 0.02, *n* = 10), and NE (*P* = 0.03, *n* = 9) (Figure 1b); whereas PAD4 (*P* = 0.07, *n* = 10) and MPO (*P* = 0.9, *n* = 10), were not statistically different (Supplementary Figure S2A). In patients with diabetes, the following NETosis markers were significantly increased after HFHD treatment compared with HDF; dsDNA (*P* = 0.03, *n* = 9), PAD4 (*P* = 0.04, *n* = 10), and NE (*P* = 0.03, *n* = 10) (Figure 1c); whereas H3 (*P* = 0.6, *n* = 10) and MPO (*P* = 0.2, *n* = 10,) markers were not significantly different (Supplementary Figure S2B). In Supplementary Figure S2, we present NETosis markers levels in healthy controls for additional context.

### Lower NETosis Markers Levels in Stimulated Neutrophils of Patients With Diabetes Compared With Patients Without Diabetes

**Figure 2:**
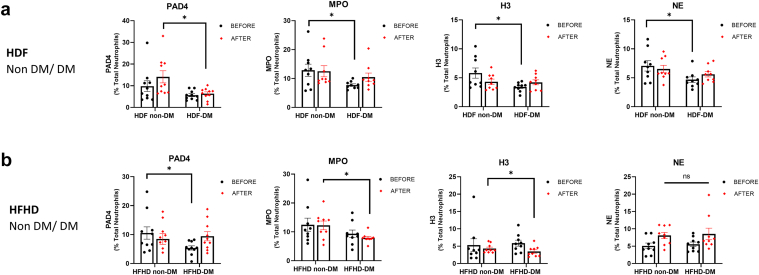
Reduced levels of NETosis markers in stimulated neutrophils of patients with diabetes compared with those without diabetes. (a) NETosis markers before and after HDF treatment in diabetic and nondiabetic participants. Patients with diabetes showed significantly lower levels of MPO, NE, and H3 before HDF than those without diabetes, and lower PAD4 levels after HDF. (b) NETosis markers before and after HFHD treatment in diabetic and nondiabetic participants. Patients with diabetes had significantly lower MPO and H3 levels after HFHD, and lower PAD4 levels before HFHD than patients without diabetes. NE levels did not differ significantly between groups. Data are presented as mean ± SEM. Statistical analysis was performed using an unpaired *t* test, ∗*P* < 0.05. H3, histone H3; HDF, hemodiafiltration; HFHD, high-flux hemodialysis; MPO, myeloperoxidase; NE, neutrophil elastase.

Next, we compared NETosis formation following neutrophils stimulation, between patients with diabetes and those without patients. Before HDF treatment, patients with diabetes exhibited significantly lower levels of NETosis markers than those without diabetes, including MPO (*P* = 0.03, *n* = 9), H3 (*P* = 0.02, *n* = 9), and NE (*P* = 0.03, *n* = 9). Following HDF treatment, patients with diabetes showed significantly lower levels of the PAD4 marker than those without diabetes (*P* = 0.02, *n* = 10) (Figure 2a). Although not statistically significant, patients with diabetes displayed a trend toward lower levels of the dsDNA marker before HDF treatment compared with those without diabetes (*P* = 0.09, *n* = 10, Supplementary Figure S3A).

After switching to HFHD, patients with diabetes continued to exhibit significantly lower NETosis markers than those without diabetes following HFHD treatment, including MPO (*P* = 0.03, *n* = 10) and H3 (*P* = 0.03, *n* = 9) (Figure 2b). Before starting HFHD treatment, significantly lower levels of the PAD4 marker were noted in patients with diabetes than in those without diabetes (*P* = 0.03, *n* = 10) (Figure 2b), whereas NE (*P* = 0.8, *n* = 9) and dsDNA (*P* = 0.27, *n* = 10) were not significantly different between the 2 groups (Supplementary Figure S3B).

Overall, patients with diabetes consistently exhibited reduced NETosis activation compared with those without diabetes, irrespective of the dialysis modality. In Supplementary Figure S3, we present NETosis markers levels in healthy controls for additional context.

### Significant Increase in Serum NETosis Markers Following 3 Weeks of HFHD

**Figure 3:**
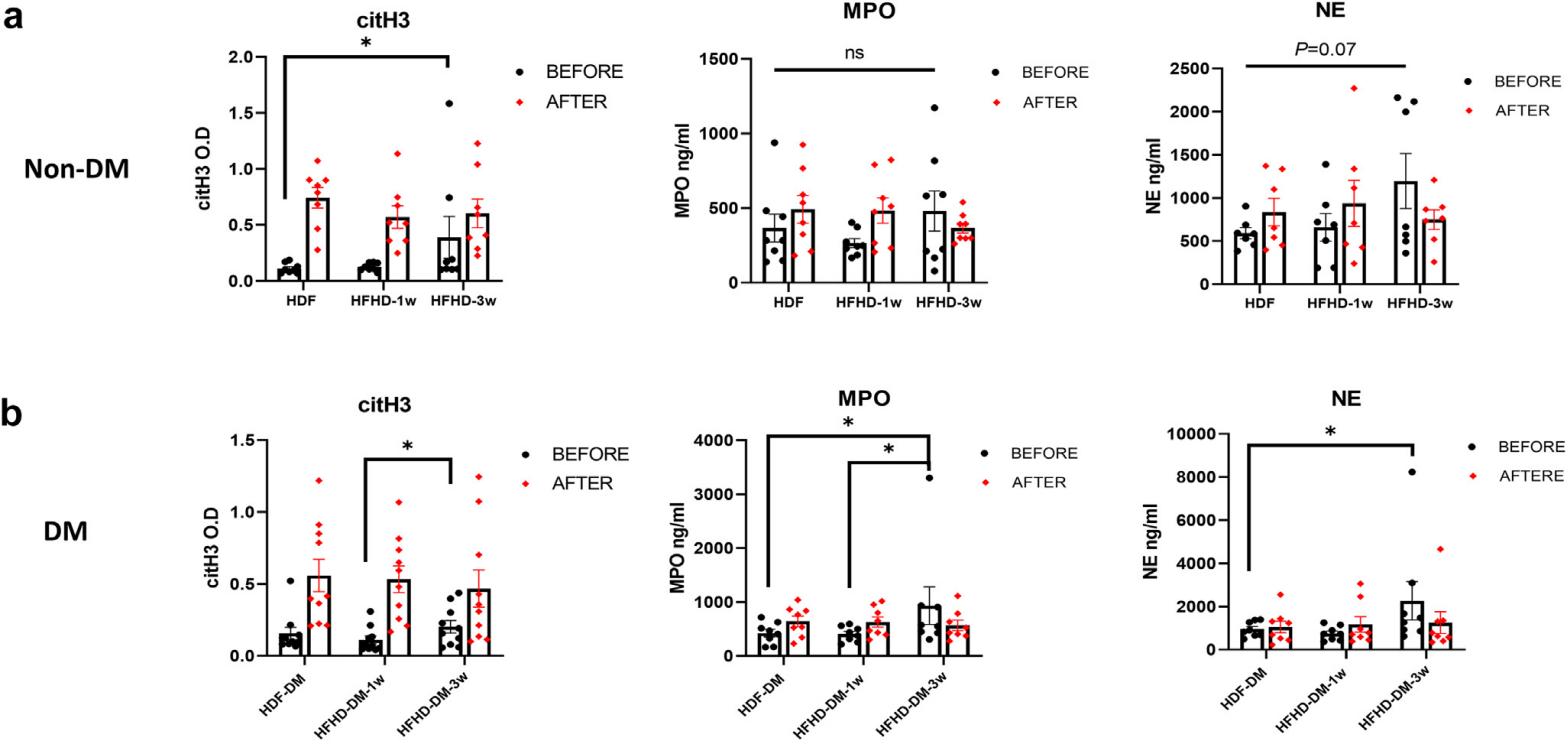
Significant increase in NETosis markers levels following 3 weeks of HFHD. Serum samples were collected before and after HDF, HFHD, and following 3 weeks of HFHD. (a) Elevated NETosis markers in serum after 3 weeks HFHD treatment in patients without diabetes. In patients without diabetes, citH3 levels were significantly elevated before HFHD treatment after 3 weeks compared with baseline levels before HDF. NE and MPO levels also increased, though not significantly. (b) Elevated NETosis markers in serum after 3 weeks HFHD treatment in patients with diabetes. In patients with diabetes, citH3, MPO, and NE levels were significantly increased in serum before dialysis after 3 weeks of HFHD. All markers were quantified using enzyme-linked immunosorbent assay. Data are presented as mean ± SEM. Statistical analysis was performed using a paired *t* test or Wilcoxon test for nonparametric data, ∗*P* < 0.05. citH3, citrullinated histone H3, HDF, hemodiafiltration; HFHD, high-flux hemodialysis; MPO, myeloperoxidase; NE, neutrophil elastase.

After converting from HDF to HFHD treatment, serum samples were collected after 1 week and again after 3 weeks of HFHD treatment. In patients without diabetes treated with HFHD for 3 weeks, there was a significant increase in citH3 marker before HFHD treatment compared with its measured levels before HDF treatment (*P* = 0.02, *n* = 8). An elevated but not statistically significant increase was observed in NE (*P* = 0.07, *n* = 8) and MPO (*P* = 0.4, *n* = 8) markers (Figure 3a). In patients with diabetes, the pattern of increased NETosis was also evident after 3 weeks of HFHD treatment. Notably, there was a significant increase in all predialysis NETosis markers 3 weeks after switching to HFHD, including citH3 (*P* = 0.04, *n* = 10), MPO (*P* = 0.02, *n* = 8), and NE (*P* = 0.04, *n* = 8) (Figure 3b). This finding indicates that, as with patients without diabetes, those with diabetes experienced enhanced neutrophil activation following prolonged HFHD treatment.

### Lower Serum Levels of citH3 in Patients With Diabetes Compared With Those Without Diabetes

**Figure 4:**
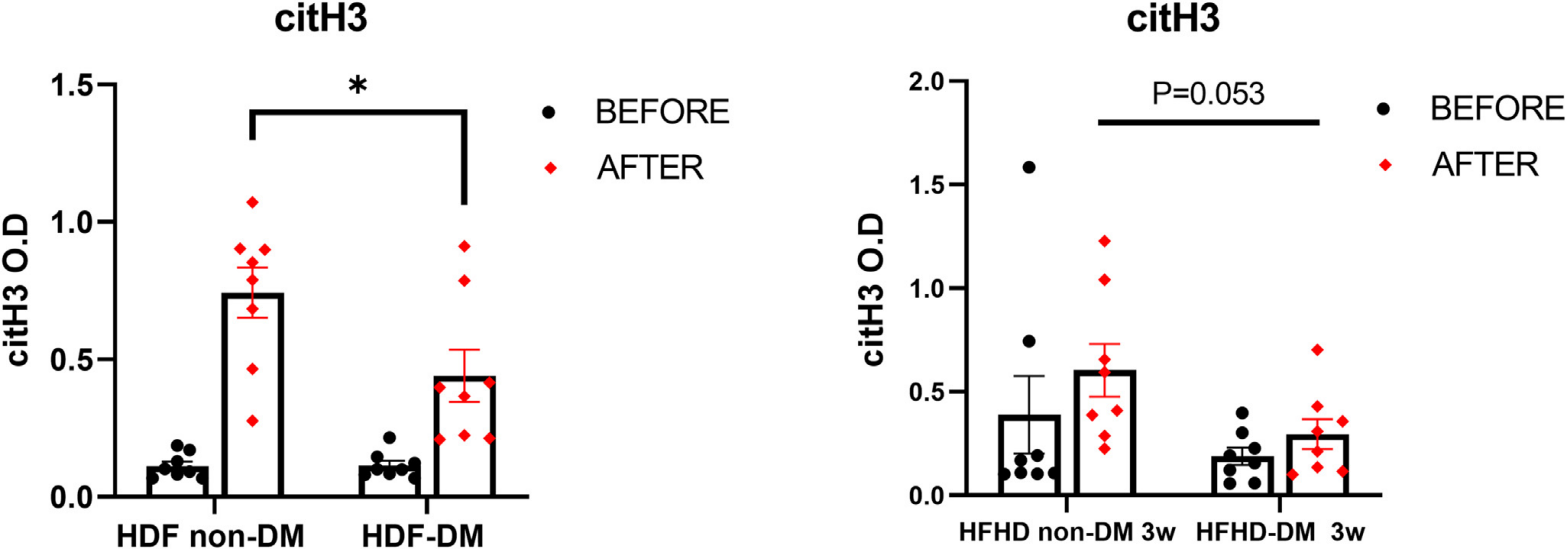
Lower serum levels of citH3 marker in patients with diabetes compared with those without diabetes. Patients with diabetes showed significantly lower serum citH3 levels than those without diabetes following HDF treatment (left panel). A similar trend of reduced citH3 levels was observed after 3 weeks of HFHD treatment (right panel). Data are presented as mean ± SEM. Statistical analysis was conducted using a *t* test, ∗*P* < 0.05. citH3, citrullinated histone H3, HDF, hemodiafiltration; HFHD, high-flux hemodialysis

Next, we sought to determine whether patients with diabetes exhibit lower serum levels of the citH3 NETosis marker than patients without diabetes, consistent with previous findings of reduced NETosis markers in stimulated neutrophils from patients with diabetes, regardless of dialysis modality. Following HDF treatment, patients with diabetes demonstrated significantly lower citH3 levels than those without diabetes (*P* = 0.04, *n* = 8). Similarly, 3 weeks after HFHD treatment, citH3 levels remained lower in patients with diabetes than in those without diabetes, though this difference approached but did not reach statistical significance (*P* = 0.05, *n* = 8) (Figure 4). These findings of lower serum citH3 levels in patients with diabetes align with earlier observations of reduced NETosis markers in stimulated neutrophils in patients with diabetes versus patients without diabetes, thus further emphasizing that patients with diabetes exhibit lower NETosis activity irrespective of the dialysis modality.

## Discussion

HD is known to exert a strong proinflammatory effect, leading to an inflammatory response with neutrophil activation and the formation of NETs.^14^ Our study aimed to investigate the impact of HD modalities (HDF vs. HFHD) on NETosis markers in both patients with diabetic and those without diabetes. Our findings demonstrate a significant increase in NETosis markers in stimulated neutrophils after HFHD treatment compared with HDF in both patient groups, highlighting the influence of dialysis modality on neutrophil activation and NETosis formation. The increased NETosis observed after HFHD compared with HDF could be attributed to HDF's superior clearance of middle molecules through convective transport, which may more effectively remove proinflammatory cytokines, thereby reducing neutrophil activation and enhancing hemodynamic stability.^14^ These findings align with previous studies suggesting that HDF may offer better biocompatibility and reduced inflammation compared with conventional HFHD.^12^, ^13^, ^14^, ^15^, ^16^, ^17^

The significant increase in serum NETosis markers after 3 weeks of HFHD treatment, compared with levels during HDF, underscores the role of HDF in attenuating dysregulated NETosis. The continued increase in NETosis markers over several weeks of HFHD treatment suggests cumulative neutrophil activation and sustained tissue injury, particularly in the vasculature, where NETs have been implicated in thrombosis and endothelial dysfunction.^6^^,^^7^^,^^10^ As previously reported, elevated NETosis markers in patients with CKD have been associated with increased cardiovascular morbidity and mortality, primarily through mechanisms involving endothelial dysfunction and accelerated atherosclerosis.^8^, ^9^, ^10^, ^11^, ^12^^,^^14^ Prolonged exposure to NET components may also contribute to organ damage by triggering the coagulation pathway and nonspecific immune system activation, which can perpetuate a cycle of inflammation and tissue injury. Furthermore, persistent neutrophil activation may lead to immune exhaustion, leaving patients more susceptible to infections, a common and serious complication in dialysis populations.^7^^,^^10^ The beneficial effects of HDF on NETosis, as evidenced by lower NETosis marker levels than with HFHD, suggest that mitigating dysregulated NET formation may contribute to the improved outcomes associated with HDF. This reduction in NETosis could potentially lead to better vascular integrity and lower the risk of complications such as thrombosis, infection, and organ damage, ultimately contributing to the lower mortality rates observed among patients on HDF.^16^^,^^17^

### Differential NETosis Activation in Patients With Diabetes and Those Without Diabetes

Interestingly, the study demonstrated lower NETosis activation and reduced levels of NETosis markers in patients with diabetes than in patients without diabetes, both before and after dialysis treatments, regardless of the HD modality. Patients with diabetes showed a trend toward shorter dialysis vintage, which could influence NETosis, although this variable was not statistically significant and is unlikely to fully account for the reduced NETosis observed in patients with diabetes. Patients with diabetes had a higher prevalence of cardiovascular disease, lower HDF substitution volume, and increased neutrophils levels. All these factors could potentially enhance NETosis, compared with patients without diabetes. The observed attenuation of NETosis in patients with diabetes is unexpected, because diabetes mellitus is widely associated with increased systemic inflammation, oxidative stress, and innate immune activation. In addition, the diabetic subgroup in our cohort demonstrated a higher prevalence of cardiovascular comorbidities, including ischemic heart disease and congestive heart failure, which are known to promote NETs formation. These clinical features would typically predict heightened NETosis activity. However, NETosis markers were consistently reduced in patients with diabetes than in their nondiabetic counterparts, across both dialysis modalities and timepoints. This paradoxical finding may indicate intrinsic alterations in neutrophil function or impaired NET-forming capacity in diabetes, rather than a response mediated solely by comorbid conditions. Notably, the within-subject design of this study, whereby each participant served as their own control during the transition from HDF to HFHD, minimized the influence of fixed variables, thus strengthening the robustness of this observation. Several hypotheses may explain this phenomenon, including the possibility of neutrophil exhaustion due to prolonged exposure to a hyperglycemic and inflammatory milieu, which could blunt the acute activation of neutrophils and reduce their capacity for NET formation. In addition, impaired glucose uptake or utilization by diabetic neutrophils may affect their ability to generate NETs. Alternatively, this reduced NETosis may reflect an adaptive response to chronic inflammation in diabetes, potentially serving as a protective mechanism against excessive tissue damage caused by persistent NET formation. Supportive evidence for the reduced NETosis in diabetes was recently reported.^18^ This study demonstrated that hyperglycemic conditions prime neutrophils and constitutively activate NETosis, leading to a reduced response to subsequent external stimuli. The authors used lipopolysaccharide as a NETosis stimulator and found that neutrophils extracted from patients with diabetes exhibited a diminished NETosis response than those from individuals without diabetes. In addition, the reduced NETosis in diabetic neutrophils was linked to lower bactericidal activity, which could only be attenuated by DNase treatment in neutrophils from healthy individuals, but not in diabetic neutrophils. This suggests that primed neutrophils in diabetes may be less responsive to additional stimulation, potentially compromising their capacity to effectively clear pathogens with increased susceptibility to infection.^18^ The chronic primed state of neutrophils in diabetes could potentially explain the reduced NETosis response observed in our study and may have clinical implications, particularly regarding infection risk in patients with diabetes who are on dialysis.

Interestingly, the subanalysis of the CONVINCE trial, which evaluated the potential benefits of HDF in patients on dialysis, did not demonstrate a statistically significant advantage of HDF in patients with diabetes. While acknowledging the inherent limitations of subanalyses, these findings are consistent with our results, suggesting that the reduced NETosis observed in patients with diabetes compared with those without diabetes may limit the incremental benefits of further NETosis attenuation via HDF. In contrast, in patients without diabetes, who exhibit a more pronounced NETosis response, HDF’s ability to moderate excessive NET formation may confer more discernible clinical benefits. This observation prompts a crucial question regarding the optimal degree of NETosis in dialysis patients. Attenuated NETosis, as seen in patients with diabetes, may increase susceptibility to infections because of compromised neutrophil function. Conversely, elevated NETosis, particularly noted in patients without diabetes following HFHD treatment, may contribute to necroinflammation and organ damage, exacerbating cardiovascular complications and morbidity. Thus, achieving an appropriate balance in NETosis modulation is imperative.

Our study has several limitations, including a single-center design, a small sample size, and a short follow-up period. Although our study design aimed to minimize interindividual variability by initiating all patients on their ongoing HDF regimen, this approach may limit the generalizability of our findings. Focusing exclusively on patients initially treated with HDF precludes full consideration of baseline differences in individuals previously managed with HFHD or other dialysis modalities.

Furthermore, though healthy controls were included to provide a qualitative reference for NETosis marker levels in nondialysis individuals, they were not fully matched for age and sex. Future studies should include larger, demographically matched control groups to better define normative ranges. Although the cohort was balanced by sex-specific analyses, gender-specific analyses were not performed because of the limited sample size. In addition, no data on hard clinical end points, such as cardiovascular events or infections, were collected because of the short intervention period. Further studies including diverse patient populations and longer observation periods are needed to validate and extend our findings.

The variability in NETosis over time and the influence of potential confounding factors may have impacted our findings. To mitigate these challenges, we employed a within-subject design, wherein each patient transitioned from HDF to HFHD, serving as their own comparator. This approach minimized the impact of individual clinical variables that could affect NETosis. In addition, the 3-week study design aimed to limit the influence of evolving clinical variables that might otherwise complicate data interpretation. Another limitation was our inability to consistently achieve the target of 23 liters of convection fluid, which has been associated with improved clinical outcomes in patients on HDF, as highlighted in the CONVINCE trial.^17^ The suboptimal convective dosing in our cohort may have attenuated the full potential of HDF to reduce NETosis. We also acknowledge that approximately 50% of participants were dialyzed via central venous catheters, which are known to be associated with increased systemic inflammation and infection risk, both of which may influence NETosis independently of dialysis modality. Importantly, none of the participants experienced active infections during the study period. In addition, our within-subject design, in which each patient transitioned from HDF to HFHD while maintaining the same vascular access, helps to mitigate this confounding variable. Despite these limitations, our findings demonstrate a significant reduction in NETosis markers during HDF compared with HFHD, and consistently lower NETosis activity in patients with diabetes than in those without diabetes.

These findings may have several important clinical implications regarding dialysis modality selection. HDF may be preferable to HFHD for reducing neutrophil activation and NETosis, potentially leading to better long-term outcomes for patients on dialysis. Moreover, tailored HD strategies might be necessary for patients with diabetes and those without diabetes because of their differential NETosis responses. Further mechanistic studies are warranted to elucidate the reasons for reduced NETosis in patients with diabetes and the differential effects of HFHD and HDF on neutrophil activation. Given the proinflammatory role of NETs, strategies aimed at modulating NETosis could be explored, including optimizing dialysis modalities or using targeted antiinflammatory therapies, such as PAD4 inhibitors or DNase treatment, to reduce NET formation. In addition, multicenter longitudinal studies are needed to investigate whether NETosis markers could serve as potential biomarkers for monitoring inflammation in dialysis patients, which could ultimately translate to reduced cardiovascular complications, infections, and overall mortality.

This study highlights the distinct effects of HFHD and HDF on NETosis in patients with diabetes and those without diabetes. HFHD induced greater NETosis activation than HDF, whereas patients with diabetes consistently exhibited lower NETosis marker levels, suggesting an altered or impaired neutrophil response. Further research is necessary to investigate the differential NETosis profiles across patient populations, their association with HD modality, and the resulting clinical implications. These findings may pave the way for personalized dialysis strategies targeting dysregulated NETosis to reduce infection risks and inflammatory injury in both patients with diabetes and those without diabetes.

## Disclosure

All the authors declared no competing interests.

## Supplementary Material

Supplementary File (PDF) **Figure S1.** Flow cytometry gating strategy for NETosis quantification. **Figure S2.** Significant increase in NETosis markers in stimulated neutrophils following HFHD treatment compared with HDF in both patients with diabetes and those without diabetes. **Figure S3.** Reduced levels of NETosis markers of stimulated neutrophils in patients with diabetes compared with those without diabetes. **Table S1.** Comparison of dialysis parameters: HDF versus HFHD. STROBE Statement Checklist.

## Footnotes

Supplementary File (PDF)
**Figure S1.** Flowa cytometry gating strategy for NETosis quantification. **Figure S2.** Significant increase in NETosis markers in stimulated neutrophils following HFHD treatment compared with HDF in both patients with diabetes and those without diabetes. **Figure S3.** Reduced levels of NETosis markers of stimulated neutrophils in patients with diabetes compared with those without diabetes. **Table S1.** Comparison of dialysis parameters: HDF versus HFHD. STROBE Statement Checklist.

## References

[1] Francis A., Harhay M.N., Ong A.C.M. (2024). Chronic kidney disease and the global public health agenda: an international consensus. Nat Rev Nephrol.

[2] Bello A.K., Alrukhaimi M., Ashuntantang G.E. (2017). Complications of chronic kidney disease: current state, knowledge gaps, and strategy for action. Kidney Int Suppl (2011).

[3] Bello A.K., Okpechi I.G., Osman M.A. (2022). Epidemiology of haemodialysis outcomes. Nat Rev Nephrol.

[4] Steiger S., Rossaint J., Zarbock A., Anders H.J. (2022). Secondary immunodeficiency related to kidney disease (SIDKD)-definition, unmet need, and mechanisms. J Am Soc Nephrol.

[5] Brinkmann V., Reichard U., Goosmann C. (2004). Neutrophil extracellular traps kill bacteria. Science.

[6] Vorobjeva N.V., Chernyak B.V. (2020). NETosis: molecular mechanisms, role in physiology and pathology. Biochemistry (Mosc).

[7] de Bont C.M., Boelens W.C., Pruijn G.J.M. (2019). NETosis, complement, and coagulation: a triangular relationship. Cell Mol Immunol.

[8] Lee H.W., Nizet V., An J.N. (2021). Uremic serum damages endothelium by provoking excessive neutrophil extracellular trap formation. Sci Rep.

[9] Kim J.K., Hong C.W., Park M.J., Song Y.R., Kim H.J., Kim S.G. (2017). Increased neutrophil extracellular trap formation in uremia is associated with chronic inflammation and prevalent coronary artery disease. J Immunol Res.

[10] Jorch S.K., Kubes P. (2017). An emerging role for neutrophil extracellular traps in noninfectious disease. Nat Med.

[11] Bieber S., Muczynski K.A., Lood C. (2020). Neutrophil activation and neutrophil extracellular trap formation in dialysis patients. Kidney Med.

[12] Cristol J.P., Thierry A.R., Bargnoux A.S., Morena-Carrere M., Canaud B. (2023). What is the role of the neutrophil extracellular traps in the cardiovascular disease burden associated with hemodialysis bioincompatibility?. Front Med (Lausanne).

[13] Jean G., Hurot J.M., Deleaval P., Mayor B., Lorriaux C. (2015). Online-haemodiafiltration vs. conventional haemodialysis: a cross-over study. BMC Nephrol.

[14] Canaud B., Blankestijn P.J., Grooteman M.P.C., Davenport A. (2022). Why and how high volume hemodiafiltration may reduce cardiovascular mortality in stage 5 chronic kidney disease dialysis patients? A comprehensive literature review on mechanisms involved. Semin Dial.

[15] Locatelli F., Carfagna F., Del Vecchio L., La Milia V. (2018). Haemodialysis or haemodiafiltration: that is the question. Nephrol Dial Transplant.

[16] Maduell F., Moreso F., Pons M. (2013). High-efficiency postdilution online hemodiafiltration reduces all-cause mortality in hemodialysis patients. J Am Soc Nephrol.

[17] Blankestijn P.J., Vernooij R.W.M., Hockham C. (2023). Effect of hemodiafiltration or hemodialysis on mortality in kidney failure. N Engl J Med.

[18] Joshi M.B., Lad A., Bharath Prasad A.S., Balakrishnan A., Ramachandra L., Satyamoorthy K. (2013). High glucose modulates IL-6 mediated immune homeostasis through impeding neutrophil extracellular trap formation. FEBS Lett.

