# Supplementary material for: Hemodiafiltration Attenuates NETosis Compared With High-Flux Hemodialysis in End-Stage Kidney Disease Patients
Source: Kidney Int Rep. 2025 Jun 10;10(9):3070–80. doi: 10.1016/j.ekir.2025.06.002 (PMC12446864; doi:10.1016/j.ekir.2025.06.002)
Supplement: Supplementary File (PDF) — Figure S1. Flow cytometry gating strategy for NETosis quantification. Figure S2. Significant increase in NETosis markers in stimulated neutrophils following HFHD treatment compared with HDF in both patients with diabetes and those without diabetes. Figure S3. Reduced levels of NETosis markers of stimulated neutrophils in patients with diabetes compared with those without diabetes. Table S1. Comparison of dialysis parameters: HDF versus HFHD. STROBE Statement Checklist. [file mmc1.pdf]

	HDF-_Non-Diabetes n=10	HD-_Non-Diabetes n=10	P-value	HDF- Diabetes n=10	HD- Diabetes n=10	P-value
**Weight (Kg), Median (IQR 25-75)**	88.55 (76.5-94.1)	88.4 (76.62-94.85)	0.27**	92.4 (75.95-96.77)	92.55 (75.75-96.52)	0.59**
**UF (Liters), Mean (Std.)**	2.75 (0.80)	2.73 (0.71)	1**	3.13 (0.59)	3.12 (0.59)	1**
**UF rate ml/kg/hour, Mean (Std.)**	8.20 (1.63)	8.15 (1.36)	1**	8.81 (1.22)	8.75 (1.16)	0.74**
**QB 300 (ml/min), n (%)**	10 (100)	10 (100)		9 (90)	9 (90)	
**QB 250 (ml/min), n (%)**				1(10)	1(10)	
**QD 500 (ml(min), n (%)**	10 (100)	10 (100)		10 (100)	10 (100)	
**Systolic BP (mm Hg), Median (IQR 25-75)**	121 (109.75-137.75)	124.5 (113.25-150.25)	0.64**	155.5 (115.75-166.5)	149.5 (111-165)	0.33**
**Diastolic BP (mm Hg), Median (IQR 25-75)**	62 (46.5-75.5)	60 (50-74)	0.19**	63 (50-78.25)	54 (47.75-66.5)	0.22**
**Fluid status: w/o edema, n (%)**	10 (100)	10 (100)		8 (80)	8 (80)	
**Fluid status: Legs edema, n (%)**				2 (20)	2 (20)	
**Treatment time, 4 hours, n (%)**	8 (80)	8 (80)		10 (100)	10 (100)	
**Treatment time, 3.5 hours, n (%)**	2 (20)	2 (20)				

Supplemental Table S1. Comparison of Dialysis Parameters: HDF vs. HFHD.

UF: ultrafiltration, QB: Blood Flow Rate, QD: Dialysate Flow Rate

** Wilcoxon Signed Ranks Test

A.

No stimulation

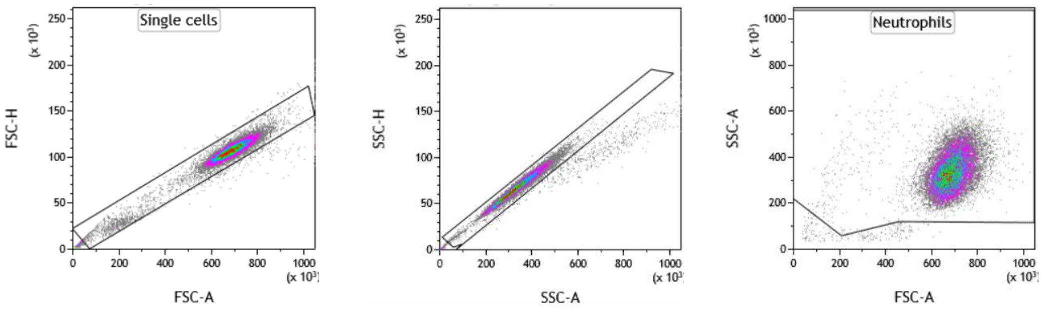

B.

100 mM PMA

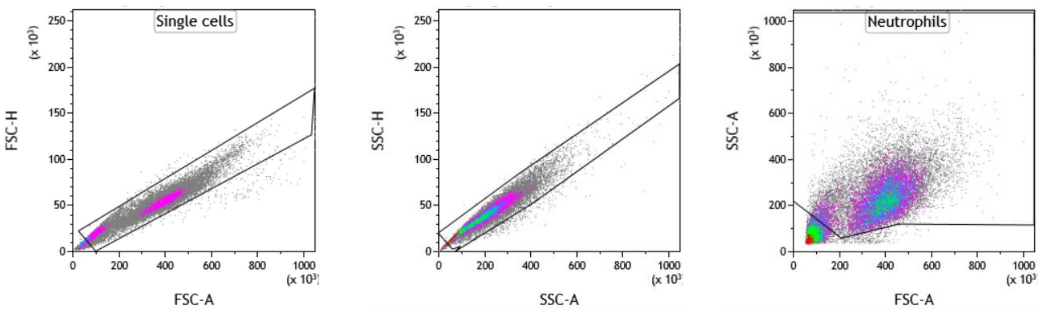

Supplemental Figure S1. Flow cytometry gating strategy for NETosis quantification. After neutrophil purification and staining for NETosis markers, all samples were analyzed by flow cytometry using a specific gating strategy. Neutrophils were gated based on forward scatter area (FSC-A) vs. forward scatter height (FSC-H) and side scatter area (SSC-A) vs. side scatter height (SSC-H), ensuring the exclusion of cell doublets and aggregates. Contaminants and debris were excluded based on their size and granularity, and analysis continued from Neutrophils population gate. **A**. Unstimulated neutrophils were used to establish NETosis baseline levels. **B**. Neutrophils treated with 100 nM PMA for 1 hour were analyzed to quantify NETosis induction.

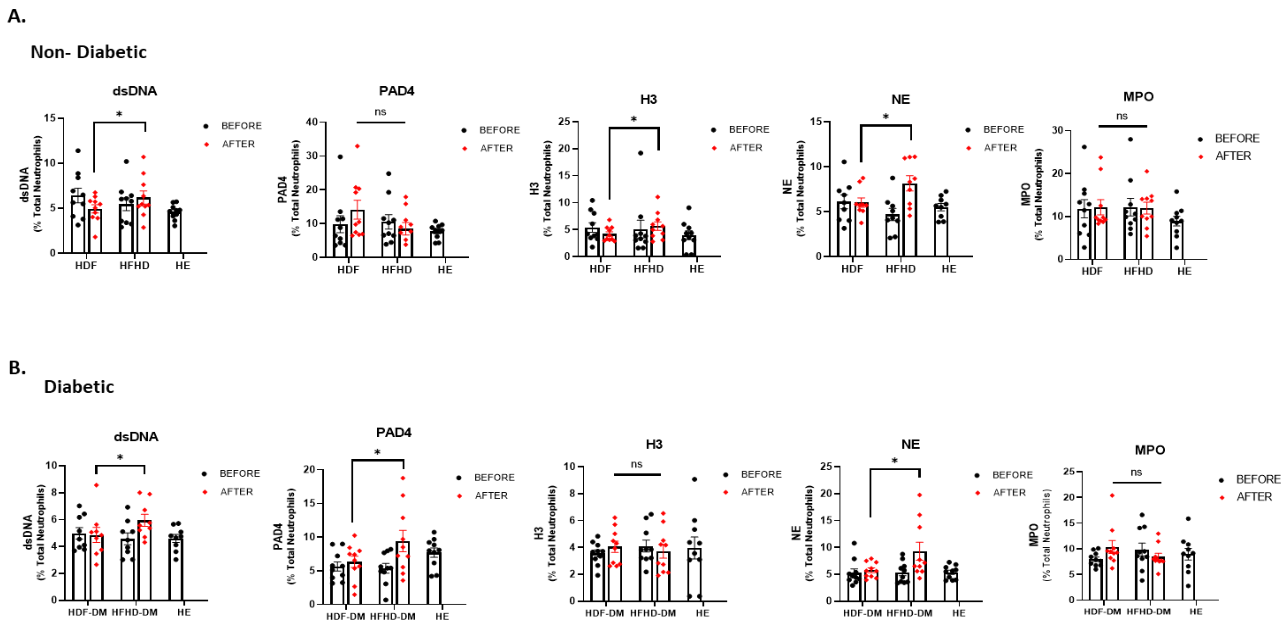

Supplemental Figure S2. **Significant increase in NETosis markers after HFHD treatment compared to HDF in both diabetic and non-diabetic patients. A**. NETosis markers in non-diabetic patients before and after HDF or HFHD treatment. Significant increases in dsDNA, H3, and NE were observed following HFHD compared to HDF. No significant change was seen in PAD4 and MPO.

B. NETosis markers in diabetic patients before and after HDF or HFHD treatment. Significant increases were noted in dsDNA, PAD4, and NE after HFHD compared to HDF. H3 and MPO levels did not differ significantly. Health controls (HE) are also presented as reference. Data are presented as mean ± SEM. All statistical analysis was performed using paired *t*-test, *P<0.05.

A.

HDF

Non DM/ DM

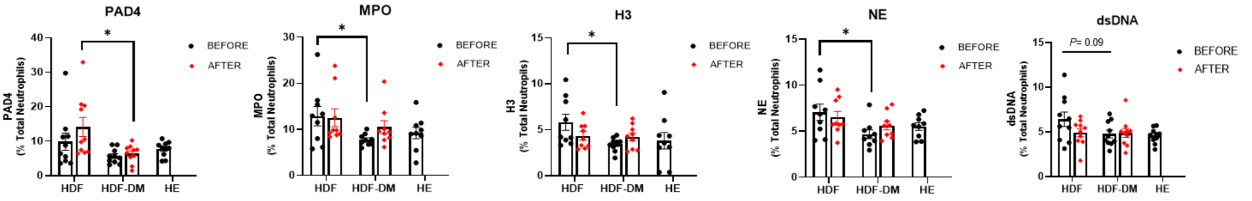

B.

HFHD

Non DM/ DM

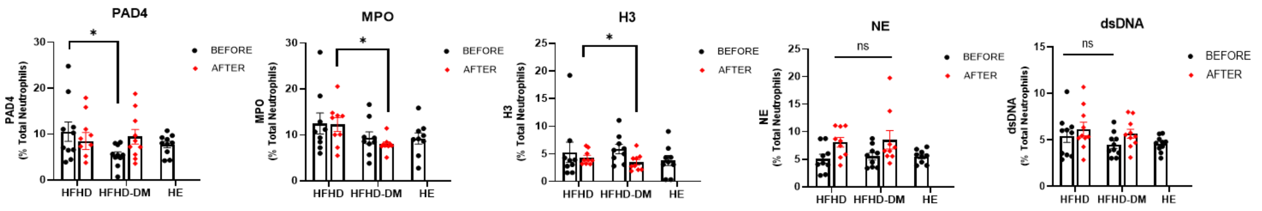

Supplemental Figure S3. Reduced levels of NETosis markers in stimulated neutrophils of diabetic patients compared to non-diabetic patients. **A**. NETosis markers before and after HDF treatment in diabetic and non-diabetic participants. Diabetic patients showed significantly lower levels of MPO, NE, and H3 before HDF compared to non-diabetics, along with a non-significant reduction in dsDNA. After HDF, PAD4 levels were also reduced in diabetic patients. **B**. NETosis markers before and after HFHD treatment in diabetic and non-diabetic participants. Diabetic patients had significantly lower MPO and H3 levels after HFHD, and lower PAD4 levels before HFHD compared to non-diabetic patients. NE and dsDNA levels did not differ significantly between groups. Health controls (HE) are also presented as reference. Data are presented as mean ± SEM. Statistical analysis was performed using an unpaired t-test, *P<0.05.

STROBE Statement—checklist of items that should be included in reports of observational studies

	Item No.	Recommendation	Page No.
**Title and abstract**	1	(*a*) Indicate the study's design with a commonly used term in the title or the abstract	1
		(*b*) Provide in the abstract an informative and balanced summary of what was done and what was found	1
**Introduction**			
Background/rationale	2	Explain the scientific background and rationale for the investigation being reported	3-5
Objectives	3	State specific objectives, including any prespecified hypotheses	5
**Methods**			
Study design	4	Present key elements of study design early in the paper	5-6
Setting	5	Describe the setting, locations, and relevant dates, including periods of recruitment, exposure, follow-up, and data collection	5
Participants	6	(*a*) *Cohort study*—Give the eligibility criteria, and the sources and methods of selection of participants. Describe methods of follow-up<br>*Case-control study*—Give the eligibility criteria, and the sources and methods of case ascertainment and control selection. Give the rationale for the choice of cases and controls<br>*Cross-sectional study*—Give the eligibility criteria, and the sources and methods of selection of participants	5-6
		(*b*) *Cohort study*—For matched studies, give matching criteria and number of exposed and unexposed<br>*Case-control study*—For matched studies, give matching criteria and the number of controls per case	
Variables	7	Clearly define all outcomes, exposures, predictors, potential confounders, and effect modifiers. Give diagnostic criteria, if applicable	6-7
Data sources/ measurement	8*	For each variable of interest, give sources of data and details of methods of assessment (measurement). Describe comparability of assessment methods if there is more than one group	6-8
Bias	9	Describe any efforts to address potential sources of bias	5-6
Study size	10	Explain how the study size was arrived at	5-6
Quantitative variables	11	Explain how quantitative variables were handled in the analyses. If applicable, describe which groupings were chosen and why	6-8
Statistical methods	12	(*a*) Describe all statistical methods, including those used to control for confounding	7
		(*b*) Describe any methods used to examine subgroups and interactions	NR
		(*c*) Explain how missing data were addressed	NR
		(*d*) *Cohort study*—If applicable, explain how loss to follow-up was addressed<br>*Case-control study*—If applicable, explain how matching of cases and controls was addressed<br>*Cross-sectional study*—If applicable, describe analytical methods taking account of sampling strategy	NR
		(*e*) Describe any sensitivity analyses	NR
**Results**			
Participants	13*	(a) Report numbers of individuals at each stage of study—eg numbers potentially eligible, examined for eligibility, confirmed eligible, included in the study, completing follow-up, and analysed	8-9
		(b) Give reasons for non-participation at each stage	NR
		(c) Consider use of a flow diagram	NR
Descriptive data	14*	(a) Give characteristics of study participants (eg demographic, clinical, social) and information on exposures and potential confounders	8-9
		(b) Indicate number of participants with missing data for each variable of interest	NR
		(c) *Cohort study*—Summarise follow-up time (eg, average and total amount)	10-12
Outcome data	15*	*Cohort study*—Report numbers of outcome events or summary measures over time	10-12
		Case-control study—Report numbers in each exposure category, or summary measures of exposure	
		Cross-sectional study—Report numbers of outcome events or summary measures	
Main results	16	(*a*) Give unadjusted estimates and, if applicable, confounder-adjusted estimates and their precision (eg, 95% confidence interval). Make clear which confounders were adjusted for and why they were included	9-12 Table1, Supplemental Table S1. Figures 1-4, Supplemental Figures S1-3

		(*b*) Report category boundaries when continuous variables were categorized	
		(*c*) If relevant, consider translating estimates of relative risk into absolute risk for a meaningful time period	
Other analyses	17	Report other analyses done—eg analyses of subgroups and interactions, and sensitivity analyses	NR
**Discussion**			
Key results	18	Summarise key results with reference to study objectives	12-16
Limitations	19	Discuss limitations of the study, taking into account sources of potential bias or imprecision. Discuss both direction and magnitude of any potential bias	15-17
Interpretation	20	Give a cautious overall interpretation of results considering objectives, limitations, multiplicity of analyses, results from similar studies, and other relevant evidence	15-17
Generalisability	21	Discuss the generalisability (external validity) of the study results	17
**Other information**			
Funding	22	Give the source of funding and the role of the funders for the present study and, if applicable, for the original study on which the present article is based	18

NR- not relevant
